# A Meta-Analysis of Tea Drinking and Risk of Parkinson's Disease

**DOI:** 10.1100/2012/923464

**Published:** 2012-02-15

**Authors:** Feng-Jiao Li, Hong-Fang Ji, Liang Shen

**Affiliations:** Shandong Provincial Research Center for Bioinformatic Engineering and Technique, Shandong University of Technology, Zibo 255049, China

## Abstract

*Background*. Many studies have reported an association between tea drinking and Parkinson's disease (PD). Our purpose is to summarize the available information and evaluate the risk of PD associated with tea drinking. *Methods*. We searched all publications in English language on the association of tea drinking and PD risk published up to December 2010. The pooled analysis was performed with Review Manager 5.0. *Results*. In total, eight articles including 1418 cases and 4250 controls were included in the meta-analysis. The pooled odds ratio (95% CI) was 0.85 (0.74–0.98), which suggests the protective effect of tea drinking in PD risks. Moreover, the summary OR (OR: 0.83, 95% CI = 0.69–0.99) for drinkers of ≤1 cup of tea per day versus nonconsumers and that (OR: 0.96, 95% CI = 0.73–1.27) for drinkers of >1 cups of tea per day versus nonconsumers showed that there was not an apparent dose-response relationship. No indication for publication bias was found. *Conclusions*. This meta-analysis showed that tea drinking can lower the risk of PD, while no apparent dose-response relationship was found. Further effort is needed to fully understand the mechanism underlying the beneficial effect of tea consumption in lowering PD risk.

## 1. Introduction

Parkinson's disease (PD) is a common neurodegenerative disorder, and, with the accelerated aging of human society, the prevalence of PD is expected to rise steadily in future [1, 2]. Despite the pathogenesis of PD is not fully elucidated, many epidemiological factors, such as coffee drinking and smoking, have been demonstrated to lower the risk of PD [3–5]. Tea is one of the most popular beverages worldwide, and the effects of tea consumption on PD risk have attracted much attention in recent years [6–17], as the tea components, such as flavonoids, caffeine, and theanine, have been proven to be neuroprotective in animal models of PD. Many studies have found that tea drinking can lower the risk of PD [6–10, 12–15], while this beneficial effect of tea is not observed in other reports [11, 16]. The purpose of the present study was to perform a meta-analysis to provide a comprehensive conclusion on the association between tea drinking and PD risk, which has important implications for the prevention and treatment of this disease.

## 2. Methods

### 2.1. Information Retrieval

We identified procurable published studies in English through a computerized MEDLINE search from 1966 to December 2010 and also the relational document links. We used the following medical subject headings and free text words to search the PubMed database: (TEA) AND (PARKINSON OR PD) AND (CASE-CONTROL OR CASE-REFERENT OR RETROSPECTIVE OR COHORT OR FOLLOW-UP OR INCIDENCE OR PROSPECTIVE OR EPIDEMIOLOG). We also examined conference proceedings; the references in the articles retrieved through the computerized search. 

### 2.2. Selection Criteria

**Figure 1:**
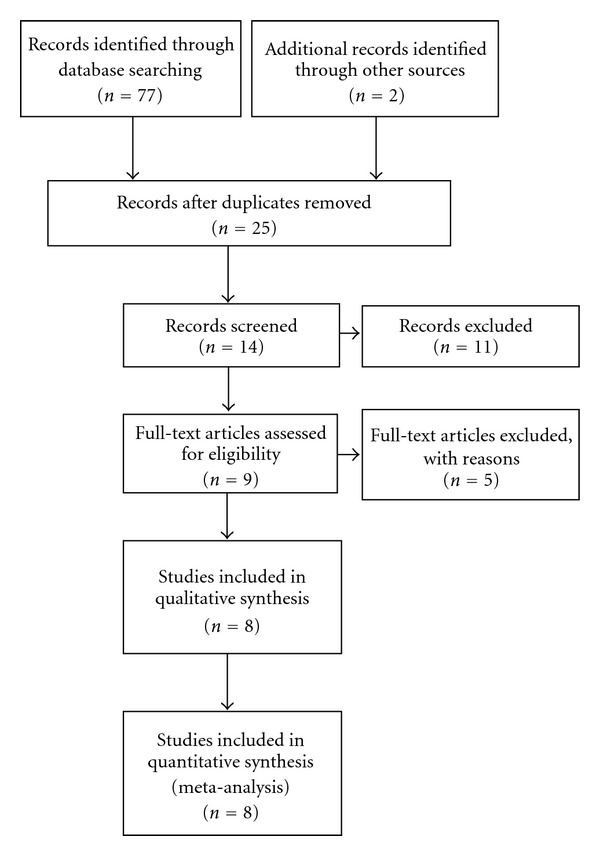
Selection of studies for inclusion in the present meta-analysis.

The identified studies must meet the following criteria: (i) the original data includes the number of PD cases studied and the odds ratio (OR) or relative risk (RR), (ii) its corresponding 95% confidence interval (CI) is for highest versus non-/lowest level of tea intake, and (iii) these studies must define the outcome of interest as incident PD based on clearly stated diagnostic criteria or identified through diagnostic codes with additional confirmation. In total, eight published articles were included in the meta-analysis [8–15]. The details of search strategy and study selection processes were shown in Figure 1.

### 2.3. Data Extraction and Meta-Analysis

We extracted information from each paper including first author, year, and country of publication, the number of cases and controls, the adjusted RRs or ORs, and 95% CI. We examined possible heterogeneity in the identified result. If the *P*  value >0.10, it means that the study does not have statistical significance. We used the fixed effect model to calculate the summary OR and its 95% CI across homogeneous studies and used the random effect model to calculate the summary OR and its 95% CI across heterogeneous studies. Egger's weighted regression method was employed to evaluate *P* value for publication bias. Finally, stratified analyses were conducted to examine the differences by cups of drinking. Statistical computation was performed using the Review Manager 5.0 statistical software.

## 3. Results

### 3.1. Tea Drinking and PD Risks

**Figure 2:**
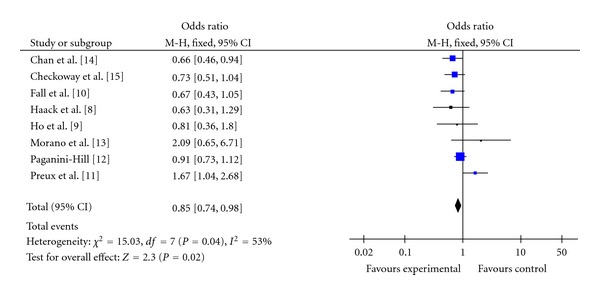
Pooled estimate of OR and 95% CI of PD risk for tea drinking. Blue diamonds indicate adjusted OR in each study. Open diamonds are pooled OR. Horizontal line represents 95% CI. Studies are ordered by alphabetical order of last name of first author.

The eight case-control studies (including 1418 cases and 4250 controls) which met the eligibility criteria were conducted in 5 countries between 1966 and 2010.  Table 1 with the extracted information present the relative risk for each of studies along with their summary OR derived from the meta-analysis.  Figure 2 presents the forest plot of comparison of tea drinkers versus non-consumers. There was statistically heterogeneity among the eight results (*P* = 0.04). The analyses compared exposed with unexposed for tea drinking, and the overall summary OR based on all studies indicated that tea drinking can protect PD (summary OR = 0.85, 95% CI = 0.85–0.98) with eight cases.

### 3.2. The Dose Effect Relationship of Tea Drinking and PD Risks

**Figure 3:**
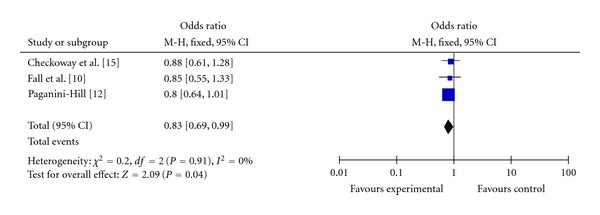
Pooled estimate of OR and 95% CI of PD risk for tea drinking of ≤1 cup per day. Blue diamonds indicate adjusted OR in each study. Open diamonds are pooled OR. Horizontal line represents 95% CI. Studies are ordered by alphabetical order of the last name of first author.

**Figure 4:**
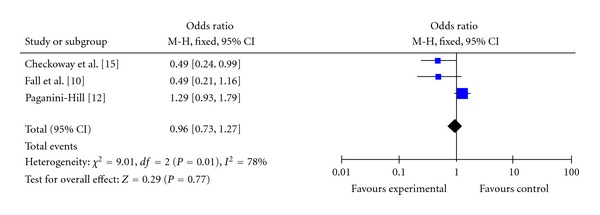
Pooled estimate of OR and 95% CI of PD risk for tea drinking of >1 cups per day. Blue diamonds indicate adjusted OR in each study. Open diamonds are pooled OR. Horizontal line represents 95% CI. Studies are ordered by alphabetical order of the last name of first author.

Among the eight studies that included in our present analysis, three reported the results for drinkers of ≤1 and >1 cup of tea per day versus non-consumers. [14, 16, 18]. The information according to the number of cups of tea drinking daily was included in Table 2. It can be seen that, for drinkers of ≤1 cup of tea per day versus non-consumers, the summary OR (OR: 0.83, 95% CI = 0.69–0.99) showed a protective effect against PD (Figure 3). For drinkers of >1 cup of tea per day versus non-consumers, the summary OR (OR: 0.96, 95% CI = 0.73–1.27) also showed a protective effect (Figure 4). According to the present results, we concluded that there was not an apparent dose-response relationship.

### 3.3. Publication Bias

**Figure 5:**
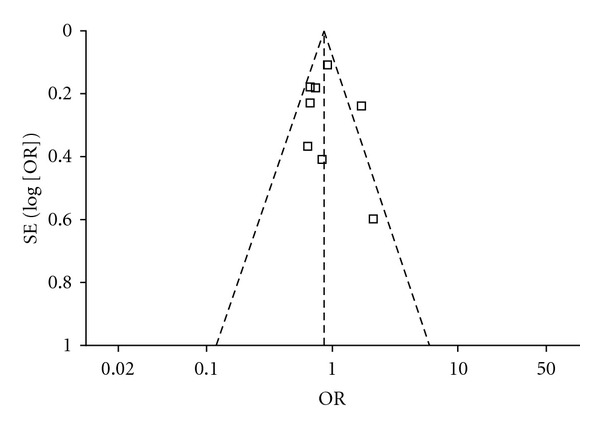
Funnel plot of tea drinking and the risk of PD. The log OR from each study is plotted on the horizontal axis, and its standard error is plotted on the vertical axis.

Figure 5 shows the funnel plot of tea drinking and the risk of PD, in which, the logOR from each study is plotted on the horizontal axis, and its standard error is plotted on the vertical axis. Inspection of the Begg funnel plot did not find the presence of publication bias because the logORs plotted against their standard errors yielded a symmetric distribution.

## 4. Discussion

As we know, PD is a common neurodegenerative disorder in middle- and old-aged crowd. The etiology of PD has long been thought to involve many factors, and there were five major hypotheses regarding the pathogenesis of PD, such as the theory of genetic factors, the theory of environmental factors, immunity doctrine, apoptosis theory, and the oxidative stress theory [18–22]. In oxidative stress theory, oxidative stress contributes to the cascade leading to dopamine cell degeneration in PD. Oxidative stress can impair altered ubiquitination and degradation of proteins directly, which have been implicated as key to dopaminergic cell death in PD, and products of oxidative damage (e.g., 4-hydroxynonenal) can damage the 26S proteasome [23].

Tea is one of the most ancient and popular beverages consumed around the world. There are many important substances in tea leaves, such as polyphenols, methylxanthine, caffeine, different lipids, amino acids, mineral substances, and volatile compounds. The demonstrated health benefits of tea include weight loss, preventing heart disease and cancer, and so forth [24]. For example, epigallocatechin gallate (EGCG), one of the most important antioxidant ingredients of green tea, is responsible for green tea's weight loss effect by increasing metabolism and the body's ability to burn fat [25]. Polyphenols in tea have been proven to inhibit tumor cell proliferation and induce apoptosis [26, 27]. Moreover, in in vitro study and experimental animal model, it has been identified that tea drinking can obviously reduce the formation of molecular products of various small lipid peroxidation and induce antioxidative enzyme activities [28].

In recent years, there were more and more studies devoted to exploring the effects of tea consumption on PD risk. Many studies reported that tea drinking can lower the risk of PD [6–10, 12–15], while this protective effect of tea is not demonstrated by other groups [11, 16]. With the aim to evaluate the association between tea drinking and PD risk, a meta-analysis was conducted based on published studies. The estimated summary OR is 0.85, which donates that tea drinking can decrease the risk of PD. This is consistent with the results of the similar study by Quintana et al. [6] and, thus, further confirms the relation of tea consumption and PD. The mechanisms underlying the protective effects of tea drinking against PD risk should be multiple. In recent years, many natural products have been proposed to be multipotent agent to combat neurodegenerative diseases [29–32]. Firstly, through synthesizing the studies in vitro and experimental animal model and the epidemiological studies we collected, we can infer that the antioxidant effect of tea components, especially polyphones, should be the predominant mechanism of tea against PD. Tea polyphones, such as EGCG, have been found to exhibit neuroprotective effect and the mechanism of the neuroprotection may involve three aspects: antioxidant, anti-inflammatory and iron-chelating activities [29, 33, 34]. Secondly, many other biological activities of tea components may contribute to the beneficial effect of tea in lowering PD risk. For instance, it was found that theanine can protect the brain by promoting the secretion of dopamine [35]. Caffeine has been demonstrated to be an adenosine A2A receptor antagonist that may enhance locomotor activity in animal models of PD and improve motor function in patients with PD [36]. In addition, Tan et al. proposed that flavonoids in tea with their anti-inflammatory effects on the cardiovascular system may help ward off PD by increasing circulation to the brain [16].

Moreover, according to the present meta-analysis, we did not find an apparent dose-response relationship as expected. It may arise from the following factors: (i) black tea and green tea differ markedly in the nature of their polyphenols [37, 38], while there were only few studies reporting stratified results according to the types of tea and (ii) the contents of the bioactive compounds in tea may fluctuate because of differences in producing areas, materials, and manufacturing [37, 38].

There were heterogeneity and limitations in our study. Firstly, the population studied in the included references was small and their location was not widespread, which make us unable to perform analysis on race. Secondly, other factors with possible influence on disease progression such as anti-Parkinsonian drugs, food supplements, or alcohol consumption were not considered. Thirdly, there were only few studies reporting stratified results according to specific types of tea (black or green) and the gender of the population studied. If more data is available in future for performing stratified analyses according to the types of tea, through comparing the components of the specific types of tea, we can identify the most important compounds, which will be of significance to the prevention and treatment of PD.

To summarize, our findings indicate that tea drinking can protect against PD, which leads human to value the impact of lifestyle on health. Despite the mechanisms behind this association should be multiple, in view of the widely accepted pathogenic role of oxidative stress in PD and the well-documented antioxidant potential of bioactive tea components, the antioxidant activity is expected to be an important aspect. Further effort is encouraged to fully understand the comprehensive mechanism underlying the beneficial effect of tea consumption in lowering PD risk.

## Figures and Tables

**Table 1:** Tea drinking and PD risk.

Author	Year of publication	Country	Cases		Controls		Relative risk	95% CI
			*N*	*n*	*N*	*n*		
Haack et al. [8]	1981	USA	237	223	474	456	0.63	[0.31, 1.29]
Ho et al. [9]	1989	China	34	21	105	70	0.81	[0.36, 1.80]
Fall et al. [10]	1999	Sweden	113	64	263	174	0.67	[0.43, 1.05]
Preux et al. [11]	2000	France	140	40	280	54	1.67	[1.04, 2.68]
Paganini-Hill [12]	2001	USA	395	182	2320	1126	0.91	[0.73, 1.12]
Morano et al. [13]	1994	Spain	74	6	148	6	2.09	[0.65, 6.71]
Chan et al. [14]	1998	China	215	113	313	196	0.66	[0.46, 0.94]
Checkoway et al. [15]	2002	USA	210	72	347	145	0.73	[0.51, 1.04]
Total			1418		4250		0.85	[0.74, 0.98]

Test for heterogeneity among all studies: *Q* = 15.03 based on 7 degrees of freedom *P* = 0.04. Summary OR was based on fixed effect models. *N*: number of cases/controls, *n*  : number of tea drinkers in cases/controls.

**Table 2:** The dose effect relationship of tea drinking and PD risk.

Author	Year of publication	Country	Cases		Controls		Relative risk	95% CI
			*N*	*n*	*N*	*n*		
Drinkers of ≤1 cup per day of tea versus non-consumers studies								
Fall et al. [10]	1999	Sweden	113	57	263	143	0.85	[0.55, 1.33]
Paganini-Hill [12]	2001	USA	395	132	2320	892	0.80	[0.64, 1.01]
Checkoway et al. [15]	2002	USA	210	61	347	110	0.88	[0.61, 1.28]
Total			718		2930		0.83	[0.69, 0.99]

Drinkers of >1 cups per day of tea versus non-consumers studies								
Fall et al. [10]	1999	Sweden	113	7	263	31	0.49	[0.21, 1.16]
Paganini-Hill [12]	2001	USA	395	50	2320	234	1.29	[0.93, 1.79]
Checkoway et al. [15]	2002	USA	210	11	347	35	0.49	[0.24, 0.99]
Total			718		2930		0.96	[0.73, 1.27]

*N*: number of cases/controls, *n*: number of tea drinkers in cases/controls.
